# Management of Strip Perforation on the Upper Front Tooth and Tooth Rehabilitation Using Flexible Glass Fiber Post: A Case Report

**DOI:** 10.7759/cureus.71950

**Published:** 2024-10-20

**Authors:** Ho Yee Mun, Rabihah Alawi, Nor Aidaniza Abdul Muttlib, Huwaina Abd Ghani, Tahir Y Noorani

**Affiliations:** 1 Conservative Dentistry, School of Dental Sciences, Health Campus, Universiti Sains Malaysia, Kota Bharu, MYS; 2 Conservative Dentistry, Hospital Universiti Sains Malaysia, Kota Bharu, MYS; 3 Prosthodontics, School of Dental Sciences, Health Campus, Universiti Sains Malaysia, Kota Bharu, MYS; 4 Prosthodontics, Hospital Universiti Sains Malaysia, Kota Bharu, MYS

**Keywords:** flexible glass fiber, iatrogenic perforation, strip perforation, tooth rehabilitation, tricalcium silicate-based cement

## Abstract

Iatrogenic perforation is caused by the degradation of the dentin root floor or wall and the overlying cementum. This condition frequently arises from improper post-space preparation, root canal negotiation and preparation, and the use of misaligned burs or mechanical instruments during endodontic access. Proper management is essential; otherwise, it could lead to the need for tooth extraction. Management of iatrogenic perforation can be challenging. In this case report, a strip perforation on an upper anterior tooth was addressed for tooth rehabilitation using tricalcium silicate-based cement and a flexible glass fiber post. This approach aims to restore the tooth's function, structural integrity, and aesthetics while minimizing complications.

## Introduction

In dentistry, iatrogenic, mechanical, or pathological crosstalk between the root canal system and the external tooth surface is termed perforation [1]. Iatrogenic perforation occurs when the dentin root floor or wall and the overlying cementum degrade as a result of improper post-space preparation, root canal negotiation and preparation, and the misaligned burs or mechanical instruments when preparing endodontic access [2]. Consequently, the periodontium becomes vulnerable to injuries, such as bone resorption, periodontal fiber damage and defects, granulomatous tissue formation, local infection and inflammation, and epithelial proliferation [3]. If the perforation is not detected promptly and treated properly, the tooth may need to be removed due to periodontium disintegration [4].

The treatment success of iatrogenic perforation depends primarily on identifying root perforations and affected sites [3]. Thus, the first step in patient management is to perform multi-angle radiographs of the root perforation and establish an accurate diagnosis [4]. Nevertheless, the diagnostic value of this method diminishes when the root perforation occurs at the buccal or palatal surfaces, as the anatomical structure might superimpose the root image [3]. Alternatively, a small field of view cone beam computed tomography (CBCT) is used to acquire the necessary information for a proper diagnosis [5]. Perforation size and site determine if the repair can be accomplished via conservative non-surgical or surgical techniques [6]. First, a dental operating microscope with high magnification is used to detect and visualize perforation along root canals [7]. Subsequently, standard perforation management is carried out by sealing the perforated tooth with bioceramic-based materials using orthograde access to prevent periradicular inflammation [8].

The root perforation is repaired and sealed correctly to deter the entry of toxic substances, which could irritate the surrounding periodontal tissues [9]. Avoiding bacterial invasion could influence the prognosis of endodontically-treated perforated teeth [6]. Ultimately, tooth repair aims to prevent inflammation and attachment loss and maintain a healthy periodontium in neighboring sites [10]. The aim of this case report was to share the management of strip perforation of the upper right central incisor (tooth 11) on a 26-year-old patient with non-surgical repair, with the assistance of a dental operating microscope.

## Case presentation

A 26-year-old female patient requested crowning on her upper front tooth. The patient had a history of a root canal treatment on tooth 11 due to pulp necrosis performed by an undergraduate student. A post and crown restoration was scheduled for this tooth, but strip perforation occurred when the student was preparing the post space. The case was then referred to a conservative postgraduate clinic for further management. On the first visit, the patient claimed the referred tooth was free of signs and symptoms and she had no known medical illness. She had never been hospitalized, was not on any prescription, and had no known food or drug allergy. She also had no history of complications or prolonged bleeding post-extraction.

**Figure 1:**
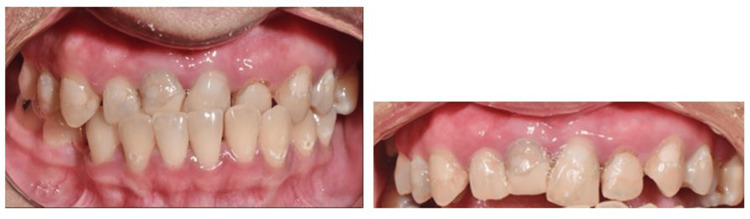
Clinical photographs of tooth 11 at initial presentation

**Figure 2:**
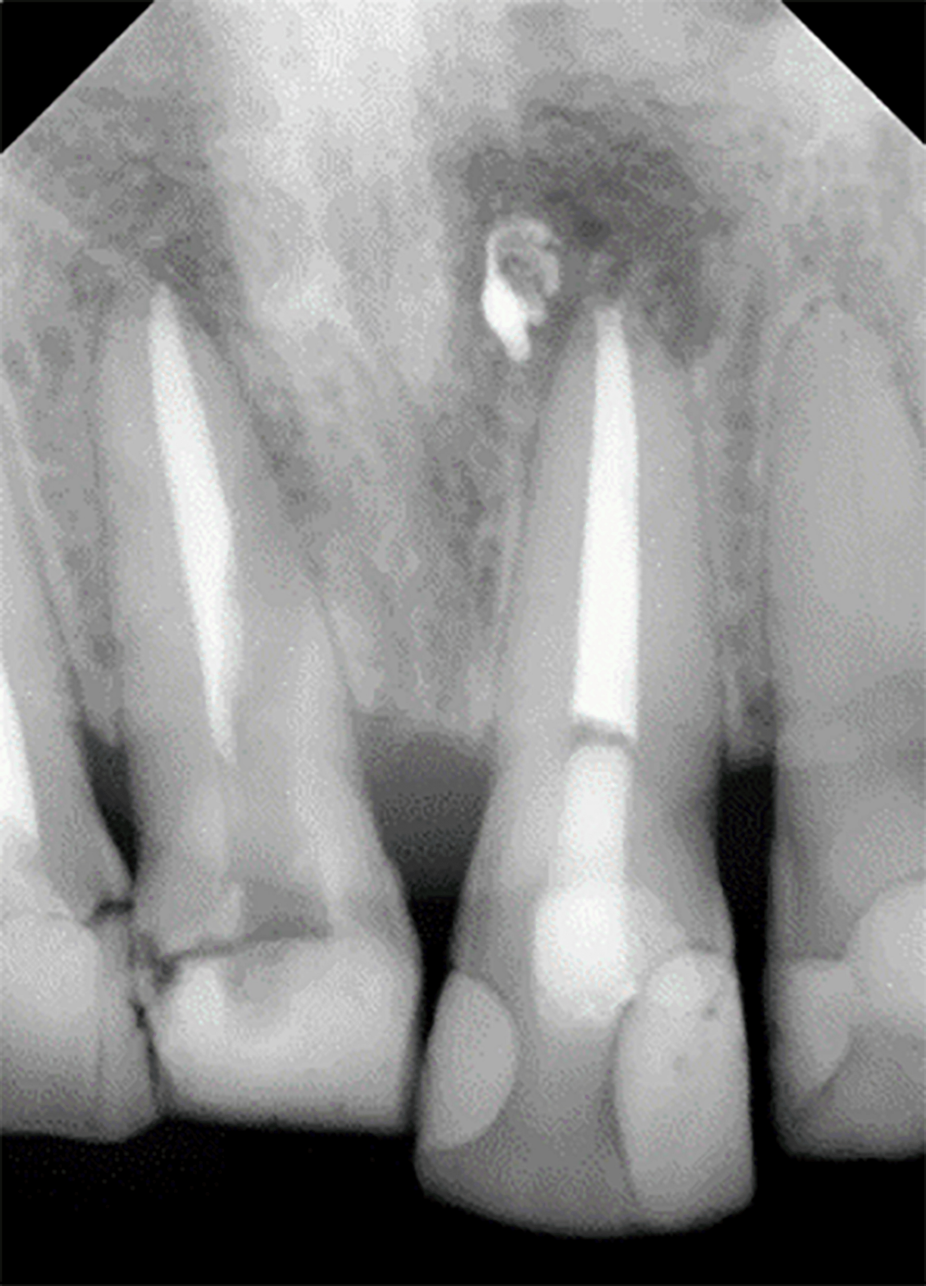
Periapical radiograph of the tooth at the initial presentation

Upon clinical examination, extensive composite and temporary restoration was observed in the patient’s tooth 11. The tooth was asymmetrical and irregular in shape and size compared to the adjacent upper central tooth. The tooth demonstrated grayish discoloration, but the surrounding soft tissues appeared normal. The clinical photograph of tooth 11 at the initial presentation is shown in Figure 1, while the radiograph of the tooth at the initial presentation is illustrated in Figure 2. The periapical radiograph demonstrated a well-condensed and apical root canal filling of tooth 11 with periapical radiolucency. Radiolucency was observed at the middle third level, mesial to the root canal. The restoration was evident at the coronal of tooth 11 due to the presence of radiopaque material. Meanwhile, radiolucency beneath the restoration margin indicated defective restoration.

**Figure 3:**
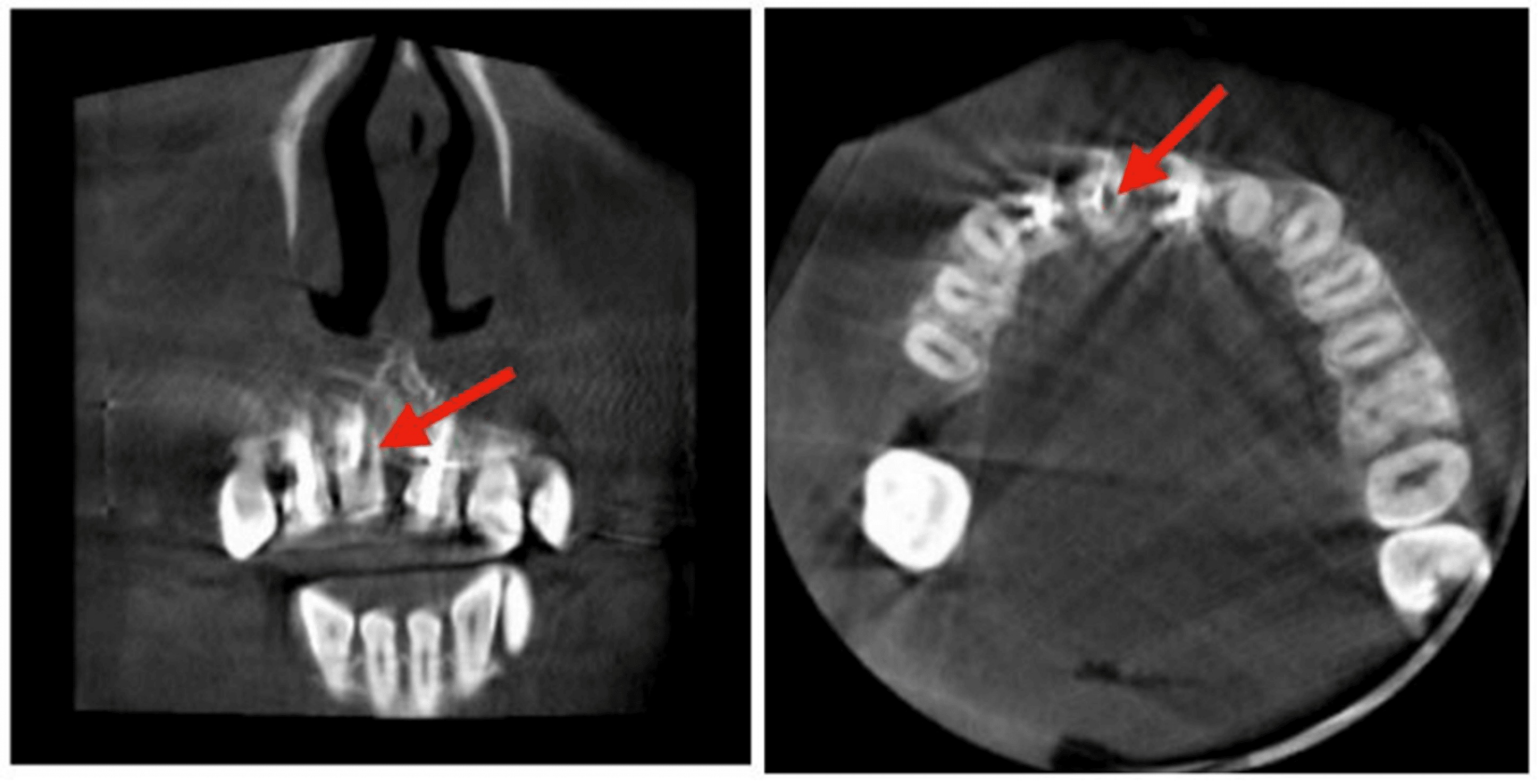
The CBCT of coronal (left) and axial planes (right) demonstrated the root perforation at the middle third level, mesial to the root canal of tooth 11 CBCT: cone beam computed tomography

A CBCT of tooth 11 was taken using Planmeca Promax 3D Mid CBCT Unit (Planmeca, Helsinki, Finland). CBCT images were viewed using Planmeca Romexis® Software (Helsinki, Finland) which revealed that the perforation occurred at the middle third of tooth 11 at the mesial site (Figure 3). A written informed consent was obtained from the patient.

**Figure 4:**
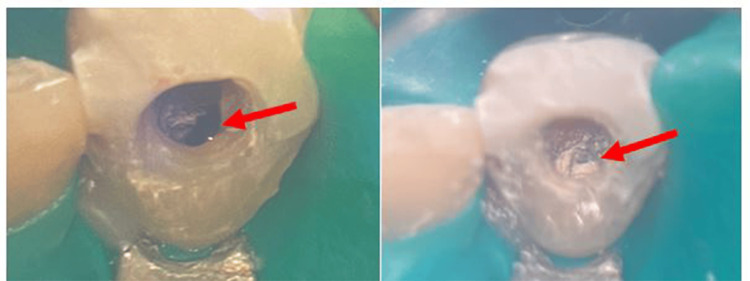
Clinical photograph of bleeding at the perforated site, mesial to the gutta-percha (GP) (left) and perforated site repaired using BiodentineTM (Septodont, France) (right)

**Figure 5:**
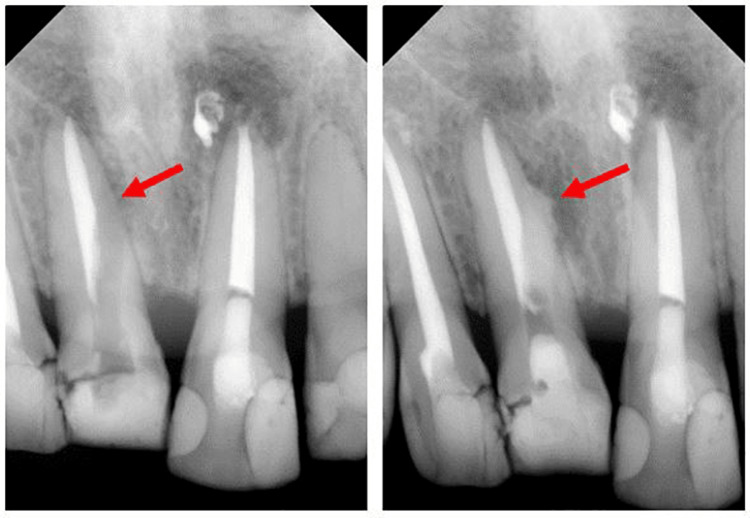
Periapical radiograph illustrates tooth 11 pre-operatively, perforated at the middle third level, mesial to the root canal (left), and perforation repair with slight BiodentineTM (Septodont, France) extrusion (right)

The existing restoration on tooth 11 was first removed with the aid of an operating microscope (Zeiss OPMI Pico, Carl Zeiss, Germany), followed by perforation repair under rubber dam isolation. When bleeding was detected at the tip of paper points (Figure 4), the perforation site was cleaned and irrigated with caution using diluted sodium hypochlorite with less concentration (2.5%) and normal saline. The perforated area was dried with paper points (size 50) before repairing the site with tricalcium silicate-based cement, BiodentineTM (Septodont, France), using the recommendation by manufacturers (Figure 4). Subsequently, the temporary double seal restoration was performed using Caviton (GC Corporation, Japan) and glass ionomer cement Fuji VII (GC Corporation, Japan). At the end of this procedure, the post-operative periapical radiograph illustrated that the perforated site had a well-condensed BiodentineTM with slight extrusion (Figure 5).

**Figure 6:**
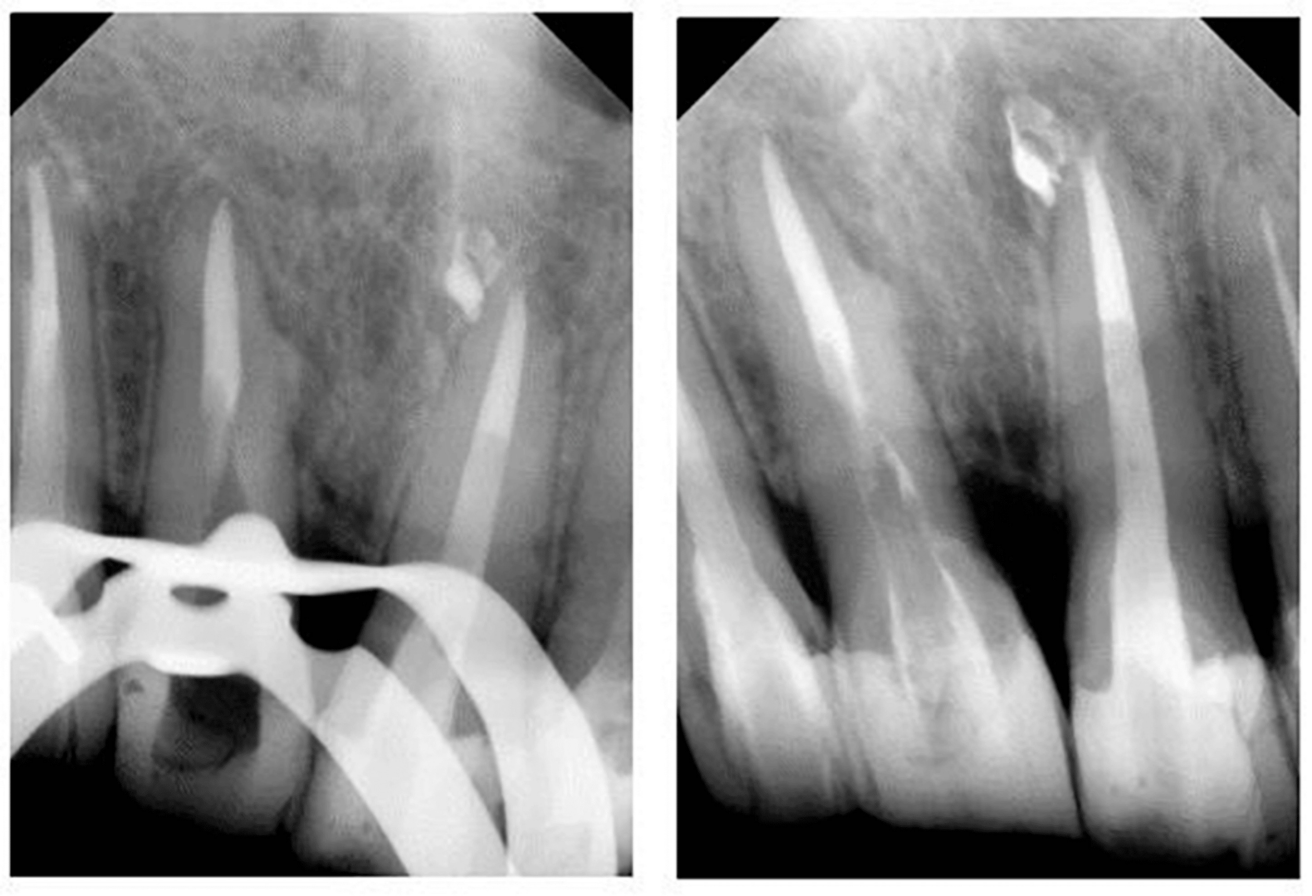
Periapical radiograph showing gutta-percha (GP) removal (left) and everStick post-cementation of tooth 11 (right)

Once the perforation was successfully repaired, the post-placement was planned for tooth 11 using the soft, flexible glass fiber everStick PostTM (GC Corporation, Tokyo, Japan) under rubber dam isolation. First, the temporary restoration and gutta-percha (GP) were removed under the operating microscope (Zeiss OPMI Pico), leaving 4 mm of GP apically followed by the preparation of the post space using the Gates Glidden bur (Figure 6). An ultrasonic instrument (Satelec ultrasonic scaler, Suprasson P5 Newtron XS, Aceton, USA) and Hedstrom files (Dentsply Maillefer, Baillage, Switzerland) were then utilized to remove the remaining BiodentineTM at the canal wall carefully. Finally, 2.5% sodium hypochlorite was used to clean and irrigate the canal, and the post length was measured using a K-file.

**Figure 7:**
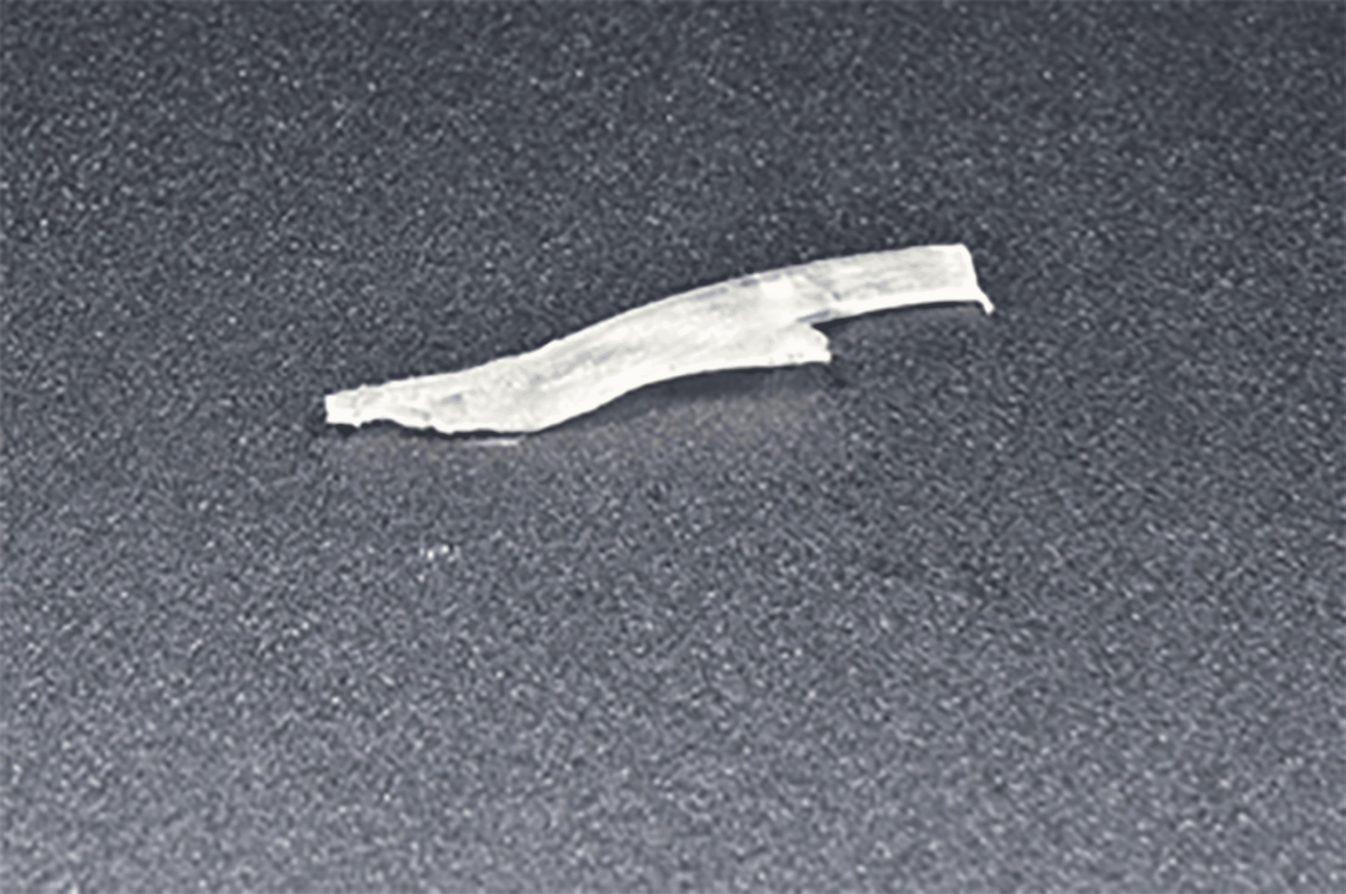
Clinical photograph of prepared everStick Post prior to cementation

After the measurement was obtained, 15 mm of the everStick PostTM (1.5 mm) was cut and fitted into the root canal. An additional 1.2 mm everStick PostTM was inserted and condensed laterally into the canal until it was completely filled. Subsequently, a periapical radiograph was taken to ensure that the post was appropriately fitted into the canal space. The next procedure was then performed by injecting a dual-cured, glass-reinforced composite system (ParaCore, Coltene/Whaledent, USA) into the post space, followed by the insertion of the prepared everStick PostTM and light-cured for 40 seconds. The post-cementation radiograph is presented in Figure 6. Figure 7 is a clinical photograph of the prepared everStick PostTM before cementation. The core was built using composite resin (3MTM FiltekTM Z350 XT Universal Restorative, 3M ESPE) and polished.

**Figure 8:**
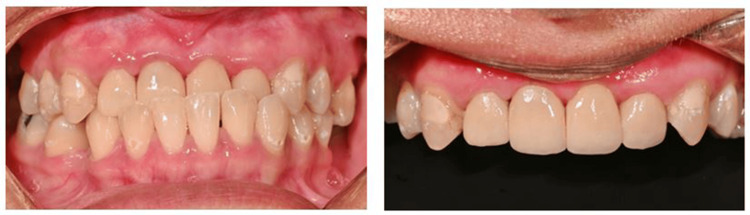
Intra-oral clinical photographs of teeth 11, 12, 21, and 22 at post-treatment

Tooth 11, together with the adjacent teeth 12, 21, and 22 were prepared for ceramic (IPS e.max Press, Ivoclar, USA) crowns using monolithic lithium disilicate. The secondary impression was established using the light and heavy body polyvinyl siloxane (PVS) (ChromacloneTM, Ultradent Products) via a stage double mix impression technique. The crowns were then fabricated in the laboratory. Subsequently, the teeth temporization was done using the putty index (3M ESPE, USA) and bisacryl composite (ProtempTM 4, 3M ESPE, USA). Zinc oxide non-eugenol temporary luting cement (FreegenolTM, GC, Japan) was used to cement the temporary crowns. Finally, the finished and polished lithium disilicate crowns were cemented using self-adhesive dual-cure resin (RelyXTM U200, 3M ESPE, Germany). The intra-oral clinical photographs of the teeth at post-treatment are presented in Figure 8.

In summary, the patient’s concerns and desire to preserve the tooth while enhancing its appearance were carefully addressed during the clinical procedure. The patient was satisfied with her improved appearance and restoration outcomes. Regular follow-up appointments have been established to monitor the patient and assess treatment success. At the six-month post-treatment review, the patient reported no complaints, and the tooth showed no signs or symptoms of complications. However, a radiograph was not taken during this appointment due to the patient's pregnancy, prioritizing her safety and well-being.

## Discussion

Perforation repair has been performed using various dental materials such as mineral trioxide aggregate (MTA), amalgam, phosphate cement, dentin chips, AH-26, intermediate restorative material (IRM), tin foil, indium foil, GP, plaster of Paris, and zinc ethoxybenzoic acid cement [8,10]. The usage of these materials over the years has recorded varying success rates. Clinicians often opt for biocompatible materials with short setting times and good sealing ability [11] to repair different types of perforations [3].

In this case, BiodentineTM, a tricalcium silicate-based cement, was selected due to its short setting time, good handling properties, easy manipulation, superior mechanical properties, and no discoloration. This biomaterial is also preferred for root perforation repair due to its biocompatibility, bioactivity, and biomineralization properties, which promote root repair and bone healing [12]. BiodentineTM also demonstrates good sealing capacity when in contact with tissue and liquid and can maintain the bone-biomaterial interface. Silva et al. [13] reported that BiodentineTM induced the formation of a biomineralizing microenvironment with minimal inflammatory response in their study, thus healing around perforations when in contact with periradicular tissues. Moreover, this biomaterial forms calcium phosphate precipitates at the material-dentine interface, potentially enhancing the tooth’s physical strength and resistance in an acidic environment [14].

One of the major steps in ensuring satisfactory patient outcomes is cleaning the perforated site thoroughly and making sure that it is well-sealed. When the perforated area is sealed properly with a biocompatible material, it is easier to maintain the health of the surrounding periodontium and improve the success rate of perforation repair [15]. In addition, the prognosis of perforation repair is linked to treatment timing and perforation site and size. For instance, an earlier study recorded higher success rates in small perforations in the coronal or apical third of the root, which were treated promptly [3]. In this case, a drill was driven in high rotation into the middle third of the root, and the perforation was completely sealed three months after the iatrogenic procedure. The prognosis in this clinical case is questionable, but recent studies have reported that perforation size, site, and timing of repair are no longer critical determinants of prognostic outcomes with the development of bioceramic materials such as MTA [8].

Perforation repair procedures also depend on the patient’s willingness to maintain their teeth, the strategic value of the teeth, and a favorable treatment prognosis [4]. The patient in this case was keen to keep her teeth and was aware of the treatment complexity, time, cost, and unpredictable longevity of the teeth. Extraction of the questionable tooth followed by implant placement may offer a promising clinical option, but there is no evidence of improved prognosis compared to natural teeth, even if the teeth have periodontal or endodontic issues [16]. Nevertheless, maintaining natural dentition longer with an appropriate recall system may delay the necessity for complex treatments [17].

The perforation on tooth 11, in this case, was repaired using BiodentineTM, and the core was supported with a soft, flexible glass fiber post or everStick PostTM. This post was selected because the individually formed, fiber-reinforced composite can be anatomically adjusted to the customized post space and prevent further loss of root dentin. This material is also easily prepared compared to cast metal or prefabricated posts [18]. Proper adaptation of the everStick PostTM can minimize stress concentration at the cement-post interface due to the thin layer of cement used [19]. The flexibility of the everStick post allows for anatomical adjustments and precisely follows the shape of the canal before light curing and attains high flexural strength after light curing. The modulus of elasticity of everStick PostTM is comparable to that of dentin, which helps reduce the risk of catastrophic root fractures. By increasing the fracture resistance and lowering the risk of unfavorable root fractures, the use of anatomically adjustable everStick posts in compromised teeth has proven to be successful [20].

This new adhesive and flexible fiber post constitutes silanated glass fibers impregnated with resin (Bisphenol-A-glycidyl methacrylate (Bis-GMA) and poly methyl methacrylate (PMMA)), forming a semi-interpenetrating polymer network (IPN) matrix after polymerization. This semi-IPN post aims to improve the bonding between the post surface and luting cement or direct composite resins to reduce adhesive failures and microleakages [18]. The enhanced bonding of individually formed fiber-reinforced composite posts with IPN resin matrix to the adhesive resin cement or composite resins is attributed to the interdiffusion bonding mechanism, which forms the “monobloc” restoration [18].

## Conclusions

The perforation repair was effectively completed in this case, where definitive restorations were performed on the tooth instead of extraction. The management of the strip perforation on the upper front tooth using a flexible glass fiber post restored the structural integrity and esthetics of the tooth. Therefore, the tooth rehabilitation performed in this case restored the patient’s dental function and esthetics. Perforation repair can still be viable, even considering the treatment’s complexity, duration, costs, and uncertain longevity of the teeth. If the patient is committed to maintaining their teeth and the teeth hold strategic value with a favorable prognosis, this approach can still be worthwhile. Maintaining natural dentition as long as possible with an appropriate recall system may delay complex treatment plans to a later time.
